# Genome-wide identification and expression analysis of the *trihelix* transcription factor family in tartary buckwheat (*Fagopyrum tataricum*)

**DOI:** 10.1186/s12870-019-1957-x

**Published:** 2019-08-07

**Authors:** Zhaotang Ma, Moyang Liu, Wenjun Sun, Li Huang, Qi Wu, Tongliang Bu, Chenglei Li, Hui Chen

**Affiliations:** 1 College of Life Science, Sichuan Agricultural University, Ya’an, China; 2 School of Agriculture and Biology, Shanghai Jiao Tong University, Shanghai, China

**Keywords:** *FtTH*, Tartary buckwheat, Development, Expression, Fruit

## Abstract

**Background:**

In the study, the *trihelix* family, also referred to as GT factors, is one of the transcription factor families. *Trihelix* genes play roles in the light response, seed maturation, leaf development, abiotic and biological stress and other biological activities. However, the *trihelix* family in tartary buckwheat (*Fagopyrum tataricum*), an important usable medicinal crop, has not yet been thoroughly studied. The genome of tartary buckwheat has recently been reported and provides a theoretical basis for our research on the characteristics and expression of *trihelix* genes in tartary buckwheat based at the whole level.

**Results:**

In the present study, a total of 31 *FtTH* genes were identified based on the buckwheat genome. They were named from *FtTH1* to *FtTH31* and grouped into 5 groups (GT-1, GT-2, SH4, GTγ and SIP1). *FtTH* genes are not evenly distributed on the chromosomes, and we found segmental duplication events of *FtTH* genes on tartary buckwheat chromosomes. According to the results of gene and motif composition, *FtTH* located in the same group contained analogous intron/exon organizations and motif organizations. qRT-PCR showed that *FtTH* family members have multiple expression patterns in stems, roots, leaves, fruits, and flowers and during fruit development.

**Conclusions:**

Through our study, we identified 31 *FtTH* genes in tartary buckwheat and synthetically further analyzed the evolution and expression pattern of FtTH proteins. The structure and motif organizations of most genes are conserved in each subfamily, suggesting that they may be functionally conserved. The *FtTH* characteristics of the gene expression patterns indicate functional diversity in the time and space in the tartary buckwheat life process. Based on the discussion and analysis of *FtTH* gene function, we screened some genes closely related to the growth and development of tartary buckwheat. This will help us to further study the function of *FtTH* genes through experimental exploration in tartary buckwheat growth and improve the fruit of tartary buckwheat.

**Electronic supplementary material:**

The online version of this article (10.1186/s12870-019-1957-x) contains supplementary material, which is available to authorized users.

## Introduction

Regulation of gene expression plays an important role in many important biological activities, such as signal transduction and cell differentiation [1]. Transcription factor (TF) binding to a specific target gene corresponding region and acting element to activate or inhibit its transcription, is a key regulatory factor that usually contains a DNA binding domain, transcriptional activation domain and nuclear localization signal [2]. More than 60 transcriptional factor families have been found in plants [1]. At present, more transcription factors, such as *MYB, NAC, WRKY,* and *AP2,* have been studied. The *trihelix TF* family is a kind of gene family that has attracted attention in recent years. This family is named for the existence of specific trihelix structures (helix-loop-helix-loop-helix) in the DNA binding domain. The domain is highly conserved among the different members of the family and is high in basic and acidic amino acids, proline and glutamine [3]. Some *trihelix* family members contain two trihelix domains, which are formed by doubling themselves from the same ancestral domain, before the ancestor differentiation of mosses and angiosperms [4]. In addition, the domain also binds specifically to GT elements on the DNA sequence; thus, the family is also known as the *GT* family [5]. The conserved domain of *trihelix* is similar to that of the three α helices (helix-helix-turn-helix) of the *MYB TF* family. Therefore, it is generally believed that the *trihelix TF* family originated from a MYB-like gene [6]. However, there are still differences in the conserved domains between the two trihelix conserved domains. The amino acid sequence between each of the two α helices of *trihelix* is longer than that of *MYB*, which leads to the difference of their three-dimensional conformation, which is why they recognize different DNA elements [6]. The *trihelix* family is widespread in terrestrial plants, although not found in green algae, and has been reported in humans and *Drosophila* [1, 7–9]. The earliest identified *trihelix* family genes are pea GT-1 protein factors that specifically bind GT elements isolated from the nucleus of pea [3]. Subsequently, genes encoding GT factors were also cloned from *Arabidopsis thaliana*, tobacco, *Glycinus*, and other plants. These factors play an important role in many physiological processes by binding with homologous GT elements or interacting with other transcription factors to regulate multiple genes [10–13]. At present, *trihelix* has been systemically identified and analyzed in many plants, including *Arabidopsis*, rice, tomato, *Brassica rapa*, *Camellia sinensis*, soybean, and *Populus trichocarpa* [5, 14–19]. Kaplan-levy et al. analyzed 30 *trihelix* transcription factors from *Arabidopsis* and 31 from rice and divided the *trihelix* family into 5 subfamilies (GT-1, GT-2, SH4, GTγ and SIP1) according to the characteristics of the conserved domain of *trihelix* [5]. All subfamilies contain N- and terminal trihelix conserved domains (except *At5g47660* in Arabidopsis), but the C-terminal domains differ. Although each subfamily contains at least one trihelix domain, there are differences between them. The internal hydrophobic region of each α helix of each trihelix domain in GT-1 and SH4 contains a tryptophan residue, while in GT-2 and GTγ, the third conserved tryptophan is replaced by phenylalanine and by isoleucine in SIP1. Members of the *trihelix* family can form a long α-helix structure at the C end, which can form a coiled-coil structure that mediates the formation of a dimer structure (some Arabidopsis and soybean *trihelix* members have been confirmed) [12, 20, 21]. Moreover, in addition to the members of SH4, the other subfamilies also contain an amphoteric α helix structure (the fourth helix); the most notable feature of GT-2 is that there is another trihelix domain at the C-terminal. Early knowledge of *trihelix* was limited to its regulation of light-dependent target genes, such as phytochrome *PhyA* [22–24]. Later, with the discovery and study of the family, it was found that *trihelix* acts a key factor in the morphological and genetic control of leaves and flowers and the development process of trichomes and embryos [25–30]. In addition, it also plays a key role in the biological stress and abiotic stress of salt stress and cold stress [14, 18, 21, 31–33].

Tartary buckwheat is one of the rare crops that can be used as both food and medicine in nature and is one of the traditional small grains in China. In the buckwheat genus, only tartary buckwheat and common buckwheat are used as food [34–36]. Tartary buckwheat contains rich flavonoids, such as rutin, quercetin, and kaempferol [37–39]. The seeds are the main sources of rutin and quercetin [40]. Rutin is a secondary metabolite that absorbs ultraviolet-b (UV-b) and protects plants from the harmful effects of UV-b radiation and disease [41–43]. Tartary buckwheat flavonoids also have many benefits for humans, such as reducing hypertension, reducing vascular permeability, acting as an anti-edema, reducing arteriosclerosis and serving as an antioxidant [44–46]. In addition, tartary buckwheat grain also contains a high balance of amino acid composition of protein, crude fiber, vitamins and starch [47–51]. A comprehensive and functional study of the *trihelix TF* family has been performed in a substantial number of plants. We have performed genome-wide studies of some *TF* families, such as *ARF, MADS, NAC and AP2* [52–55]. In contrast, the information and function regarding *trihelix* in tartary buckwheat has not yet been carried out. Because the *trihelix* gene has many important physiological functions and is important to the plant, it is of great scientific interest to systematically analyze the *Fagopyrum tataricum trihelix (FtTH) TF* family members. In 2017, Zhang et al. revealed the genomic sequence of tartary buckwheat, which helps us to study the physiological and biochemical properties and expression of genome-wide *FtTH* [56]. In this study, 31 *FtTH* genes were systematically analyzed and grouped into 5 clades according to the number of DNA binding domains, the conserved amino acids of GT protein domains and the classification of *Arabidopsis*. We provided detailed information on *FtTHs*, including physical properties, motif organization, chromosomal localization and gene replication events. Seven comparative system diagrams were set up between tartary buckwheat and seven other dicotyledonous species. Moreover, the expression characteristics of *FtTH TF* factors exhibited in specific tissues/organs (stem, root, leaf, flower and fruit) are concluded according to the result of real-time quantitative PCR (qRT-PCR). We further analyzed the temporal differences and characteristics of 28 *FtTH* genes at different fruit development stages using the same method. The whole genome identification and expression analysis of the *trihelix* family in tartary buckwheat helps to better understand the *TF* family and provide an adequate theoretical basis to further verify the function of candidate genes and improve the species.

## Methods

### Identification of the *trihelix* family in tartary buckwheat

We downloaded the entire tartary buckwheat genome sequence information using Tartary Buckwheat Genome Project (TBGP; http://www.mbkbase.org/Pinku1/). Based on two BLASTp methods, *FtTH* family members were identified. First of all, with BLASTp, all possible FtTH proteins were identified from tartary buckwheat genome referring to trihelix protein sequences of *Arabidopsis*. The Hidden Markov Model (HMM) profile consistent with the trihelix domain was obtained from Pfam protein family database (http://pfam.sanger.ac.uk/) [57]. Candidate FtTH proteins containing the trihelix domain was screened out with PFAM and SMART programs. Then, these trihelix proteins were verified whether they were all members of trihelix family with BLASTp in NCBI. In the end, we screened 31 tartary buckwheat *trihelix* genes. In addition, the basic features of the trihelix proteins identified, such as the coding sequence length (CDS) and isoelectric point (pI), were determined on the ExPasy website (http://web.expasy.org/protparam/).

### Phylogenetic analyses and classification

The *Arabidopsis* trihelix and FtTH protein sequences were used for multiple amino acid sequence alignments using Clustalx1.81 program. Through Geneious R11 by the Neighbor-Joining (NJ) method, the phylogenetic tree related to 29 *trihelix TF* family members from *Arabidopsis* and 31 from tartary buckwheat were established. Thirty-one *GT* factors were distributed in different clades based on the number of DNA binding domains, the conserved amino acids of GT protein domains and the classification of *Arabidopsis*. In addition, phylogenetic trees were constructed among tartary buckwheat, *Arabidopsis thaliana*, *Beta vulgaris*, *Solanum lycopersicum*, *Vitis vinifera*, *Oryza sativa* and *Helianthus annuus* with the same method above. These trihelix protein sequences were obtained from the UniProt database (Available online: https:/www.uniprot.org).

### Exon/intron structures and conserved motif analysis

The exon/intron structures of the *FtTH* genes were generated by the Gene Structure Display Server (GSDS: http://gsds.cbi.pku.edu.cn) online tool. To compare the differences in *FtTHs*, the conserved motifs of the trihelix proteins were determined. The analysis of these conserved protein motifs in tartary buckwheat trihelix proteins was performed with the protein conserved motif online search program MEME (http:/meme.nbcr.net/meme/intro.html). The relative parameters were set to the motif breadth as 6 to 200 amino acid (aa) and the number of motifs as 10 [52].

### Chromosomal spread, gene duplication and collinear analysis with other species

The location information of *FtTH* genes obtained by Circos showed that all *FtTH* genes were spread on tartary buckwheat chromosomes [58]. The detection and study of the gene duplication events in *FtTH* genes were performed with the use of multiple collinear scanning toolkits (MCScanX) with E-value set to 10^− 5^ [59]. The syntenic analyses on the relationship of the *trihelix* family between tartary buckwheat and seven other dicotyledonous plants (*Arabidopsis thaliana*, *Theobroma cacao, Glycinus max, Beta vulgaris*, *Solanum lycopersicum*, *Vitis vinifera*, and *Helianthus annuus*) using the Dual Systeny Plotter software (https://github.com/CJ-Chen/TBtools) [60].

### Plant materials

The tartary buckwheat accession (XIQIAO) materials used in the experiment were supplied by Professor Wang Anhu of Xichang University. From 2013 to 2018, XIQIAO was introduced into the experimental field of the College of Life Science, Sichuan Agricultural University, Ya’an, Sichuan, China, and the ecological environment and cultivation conditions were the same during those years. The materials were collected in 2018. We collected the flowers, stems, roots, leaves and fruit at three developmental stages: 13 (green fruit stage), 19 (discoloration stage) and 25 (initial maturity stage) days postanthesis (DPA) [61] of mature tartary buckwheat (XIQIAO) at different development stages from the tartary buckwheat experimental base located at the farm of Sichuan Agricultural University. We kept the collected samples at − 80 °C for subsequent experiments.

### Expression profiles for *FtTH* genes

With *FtTH* family members in the tissues collected (stems, roots, leaves, flowers, and fruit), we analyzed their expression patterns at least three times with the help of qRT-PCR analysis. The gene-specific primers for qRT-PCR determination shown in Additional file 4: Table S4 were designed from Primer3 software (http://frodo.wi.mit.edu/). The whole expression analysis was performed with the tartary buckwheat reference gene *H3* as the internal reference. SYBR Premix Ex Taq II (TaKaRa) was utilized in qRT-PCR determination. By the 2^−(∆∆CT)^ method, the final test results can be calculated to the corresponding data [62].

### Statistical analysis

Variance analysis was conducted on all the data and results with the use of the Origin Pro 2018b (OriginLab Corporation, Northampton, Massachusetts, USA) statistics program and compared with the least significant difference (LSD) at the 0.05 and 0.01 levels.

## Results

### Identification of the *FtTH* family in tartary buckwheat

In the study, we identified 31 *trihelix* genes in the tartary buckwheat genome database by two BLAST methods based on the known trihelix domain sequence of *trihelix* genes by removing redundant genes (Additional file 1: Table S1). In Additional file 1: Table S1, 31 *FtTH* genes were named from *FtTH1* to *FtTH31* according to their respective order on the chromosomes.

The physical properties and characteristics of predicted *FtTH* genes, including CDS, molecular weight (MW), isoelectric point (pI) and subcellular localization, are shown in Additional file 1: Table S1. The length of 31 FtTH proteins varied from 129 (FtTH12, FtTH17 and FtTH22) to 863 aa (FtTH6), with an average of 388 aa (Additional file 1: Table S1). They had the lowest MW of 14.70 KDa *(FtTH12)* and the highest MW of 95.75 KDa *(FtTH6)*, with an average of 43.79 KDa. The PI varied from 4.76 (*FtTH8*) to 9.53 (*FtTH7 and FtTH18),* with an average of 7.37. According to the prediction of subcellular localization (Additional file 1: Table S1), 29 *FtTH* family members may be located in the nucleus, while only 2 *FtTH* family members may be located in the chloroplast. In addition, the sequences of the identified *FtTH* genes and amino acid sequences of FtTH were provided in Additional file 1: Table S1.

### Phylogenetic analysis and classification of *FtTH* genes

**Fig. 1:**
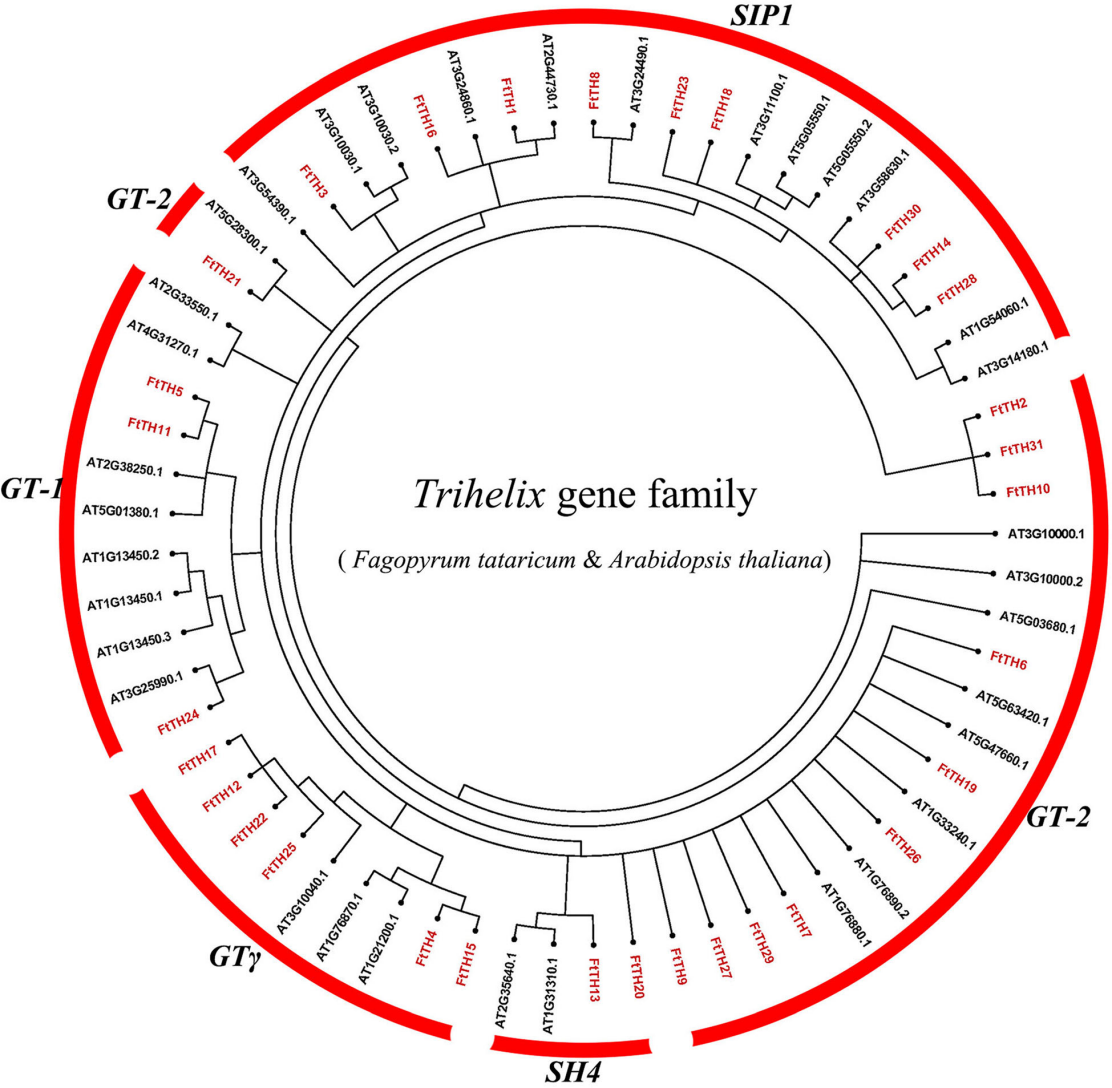
Unrooted phylogenetic tree representing relationships among 31 *FtTH* genes from tartary buckwheat and 29 *Arabidopsis thaliana trihelix* genes. 31 *FtTH* genes from tartary buckwheat are classified into group GT-1, GT-2, SH4, GTγ and SIP1. The *trihelix* genes of tartary buckwheat and *Arabidopsis thaliana* are marked in red and black, respectively

In the reported study, 30 *trihelix* genes were identified in the model plant *Arabidopsis thaliana*. To study the phylogenetic relationship of *trihelix* in tartary buckwheat and *Arabidopsis thaliana*, we chose 29 *Arabidopsis thaliana trihelix* members and 31 *FtTH* members to construct an unrooted NJ tree (Fig. 1). The phylogenetic tree of *trihelix* genes shown in Fig. 1 were grouped into 5 subfamilies (GT-1, GT-2, SH4, GTγ and SIP1) according to the number of DNA binding domains, the conserved amino acids of the GT domain and the classification in *Arabidopsis thaliana* [5]. In the phylogenetic tree, GT-2 with 11 *FtTH* family members is the largest clade. SH4 with 2 *FtTH* family members is the smallest cluster. Moreover, the numbers of clade GT-1, GTγ and SIP1 were 3, 6 and 9, respectively. Based on the results, *AT2G33550.1 and AT4G31270.1* from SH4 of *Arabidopsis thaliana* were not grouped because they were not highly homologous to any tartary buckwheat *FtTH* genes.

### Gene structural characteristics and conserved motif compositions of *FtTHs* genes

**Fig. 2:**
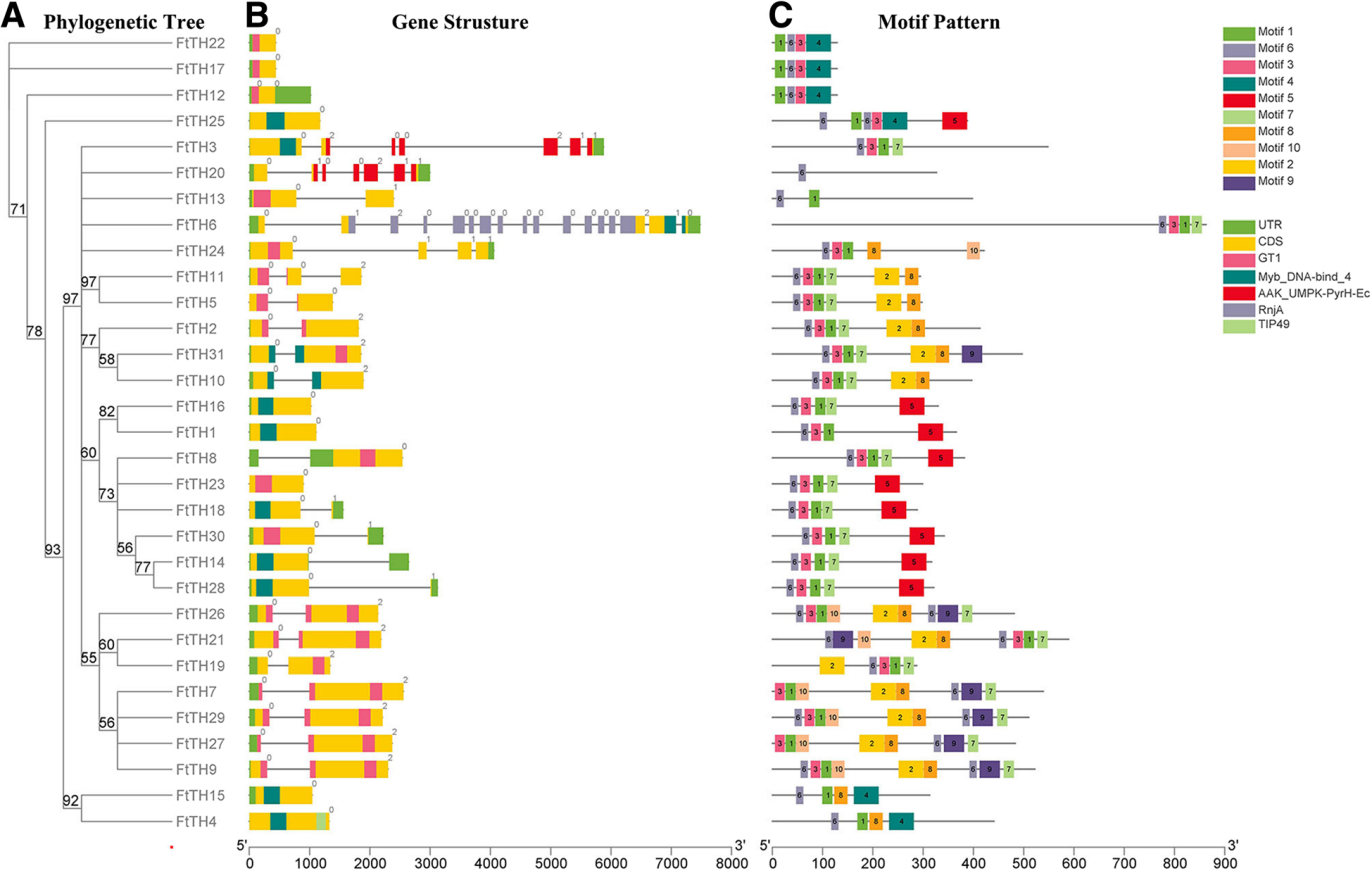
Phylogenetic relationships, gene structures and compositions of the conserved protein motifs of the *FtTH* genes from tartary buckwheat. **a.** The phylogenetic tree was constructed based on the full-length sequences of tartary buckwheat trihelix proteins using Geneious R11 software. **b.** Exon-intron structures of tartary buckwheat *trihelix* genes. Green boxes indicate untranslated 5′- and 3′-regions; yellow boxes indicate exons, and black lines indicate introns. The trihelix domain is highlighted by a pink box. The number indicates the phases of the corresponding introns. **c.** The motif compositions of the tartary buckwheat trihelix proteins. The motifs, numbered 1–10, are displayed in different colored boxes. The sequence information for each motif is provided in Additional file 2: Table S2. The protein length can be estimated using the scale at the bottom

The features of the *FtTH* gene structure are shown in Fig. 2b, including the number and distribution of exons and introns. The coding sequences (CDS) of more than half of the *trihelix* genes were separated by introns. In general, the trihelix members grouped in the same clade share similar exon/intron organizations based on the exon/intron number. By analyzing the gene structural characteristics, we determined that 13 *FtTH TF* family members had no introns, of which they were mostly from clade GTγ and SIP1. Among other *FtTH* genes, they contained 1, 2, 3, 6 or 16 introns. Of these, 13 *FtTH* genes contained 1 intron, 2 *FtTH* genes contained 6 introns, and three other *FtTH* genes contained 2, 3 or 16 introns. The number of introns in the same clade of *FtTH* genes is almost the same, except for *FtTH6*. The number of exons varied from 1 to 5, showing that there were some differences in degree among the 31 *FtTH* genes.

To further analyze the diversity of the trihelix in tartary buckwheat, the MEME search tool was used to predict 10 conserved motifs (motif1~motif10) of FtTH proteins shown in Fig. 2c. The motif organizations of every FtTH protein are shown with the corresponding color boxes in Fig. 2c. The detailed sequence of each motif is provided in Additional file 2: Table S2. Motif 1, 3 and 6 are present in almost all FtTH proteins, and all the proteins contain motif 6. Different groups shared similar motifs, suggesting that these conserved motifs might have pivotal roles in specific functions. In addition, some FtTHs have more than one motif 6, such as FtTH9, FtTH21, FtTH25, FtTH26 and FtTH29*,* which are mainly in clade GT-2. All tartary buckwheat trihelix family members in clade SIP1 share motif 1, 3, 5, 6 and 7 except FtTH3, which lacks motif 5. The conserved motif constitutions of clade GT-1 and GT-2 are superficially similar; they all possess motif 1, 2, 3, 6, 7 and 8 except FtTH19 and FtTH24. However, motif 4 was only found in clade GTγ; motif 5 existed almost exclusively in clade SIP1; motif 9 was only found in clade GT-2; two FtTHs of clade SH4 possess the least number of motifs, and interestingly, FtTH20 only has motif 6.

### Chromosomal distribution and gene duplication events of *FtTH* family

**Fig. 3:**
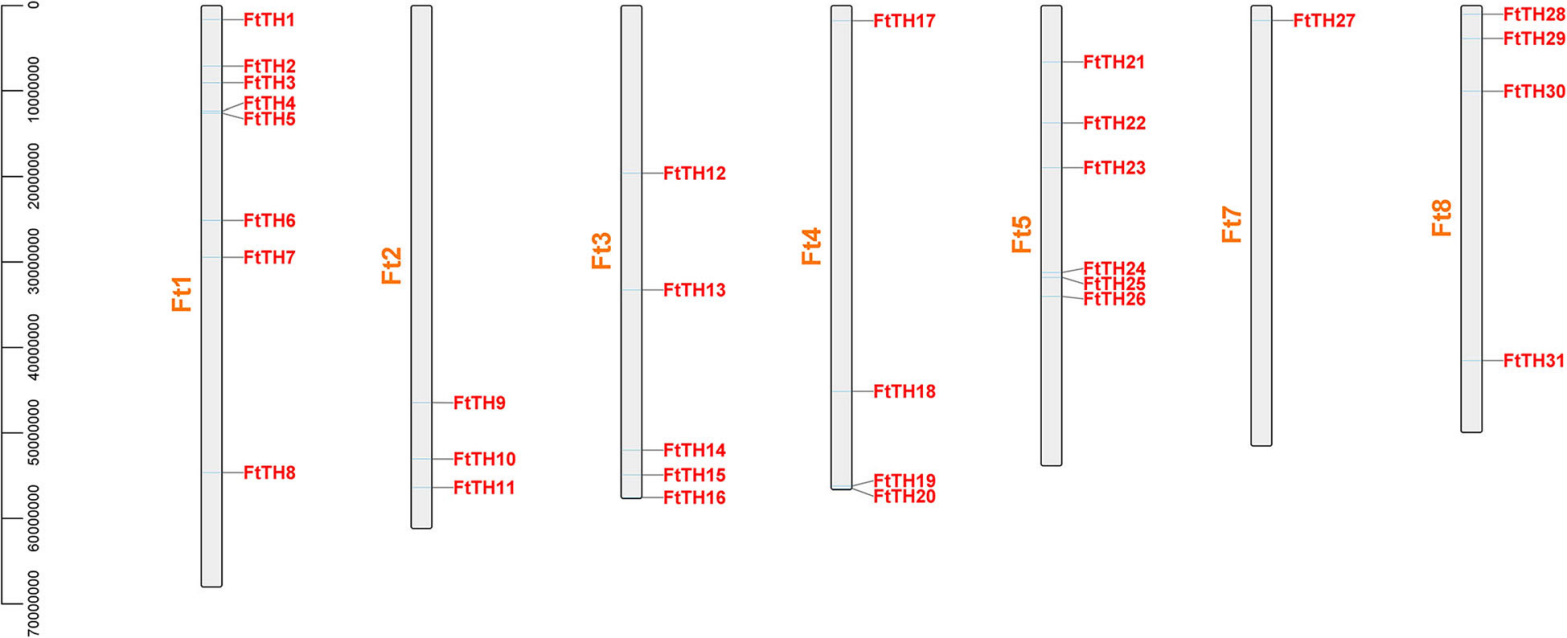
Schematic representations of the chromosomal distribution of the tartary buckwheat *trihelix* genes. The chromosome number is indicated to the right of each chromosome

**Fig. 4:**
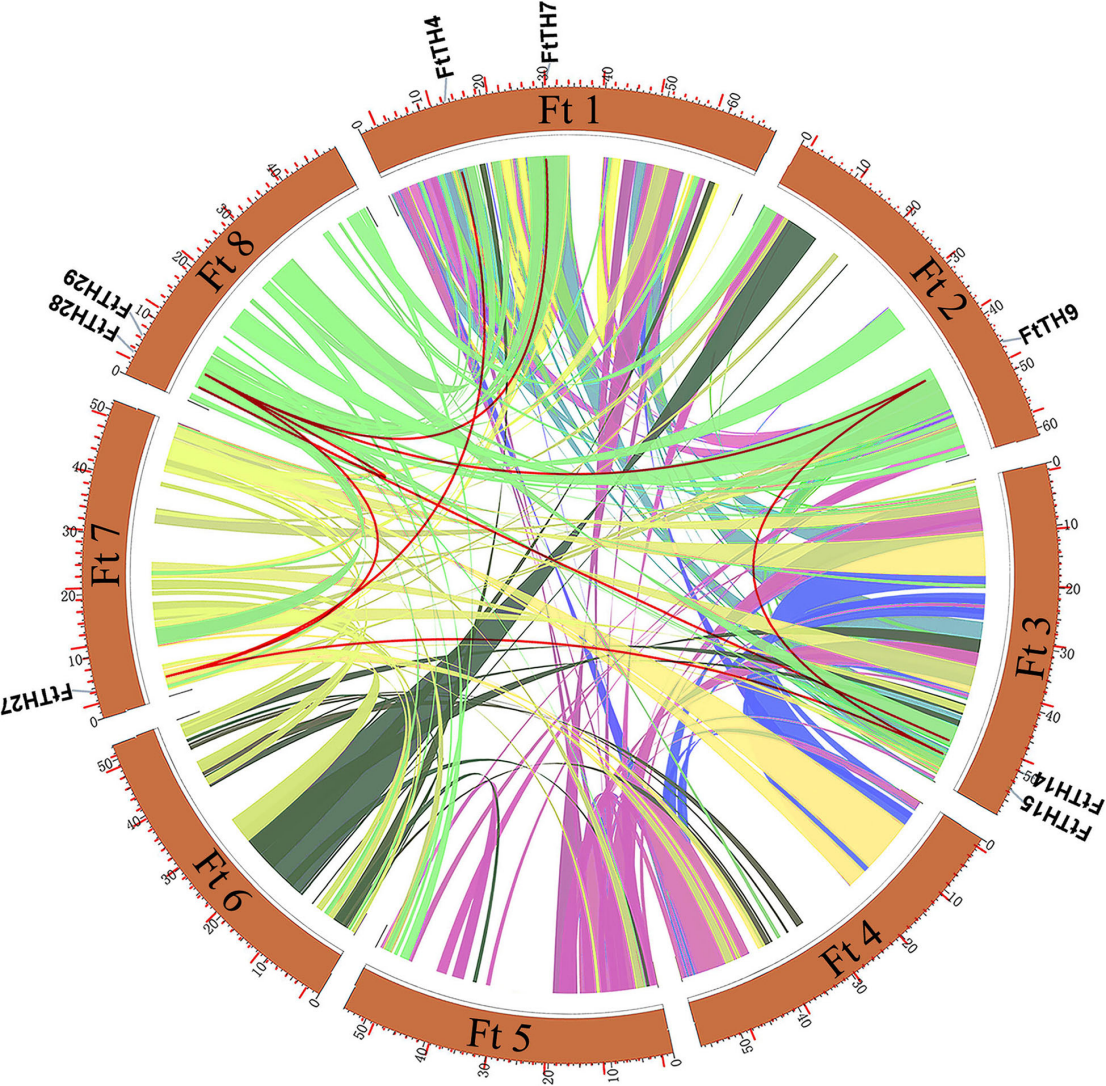
Schematic representations of the interchromosomal relationships of the tartary buckwheat *trihelix* genes. Colored lines indicate all syntenic blocks in the tartary buckwheat genome

In the chromosome map (Fig. 3), a total of 31 *FtTH* genes were dispersed in 8 chromosomes. Some chromosomes contained more *FtTH* genes. The number of *FtTH* genes on each chromosome ranged from 1 to 8, with the largest number of *FtTH* genes, which was 8 on the first chromosome. However, there were no *FtTH* genes on chromosome 6. To analyze the evolution of the *FtTH TF* family, the gene duplication events were analyzed, including tandem and segmental duplication events. Tandem and segmental duplication are key factors in enriching protein function and promoting gene evolution and expansion [63]. Using MCScanX, it was found that there were no tandem duplication events between these *FtTH* genes, and only segmental duplication exists in the *FtTH* gene family, as shown in Figs. 3 and 4. While the genes are located on 8 chromosomes of the tartary buckwheat genome, there are some pairs of repeated *FtTH* gene fragments (*FtTH29* and *FtTH7/FtTH9/FtTH14/FtTH27; FtTH15 and FtTH9/FtTH27; FtTH14 and FtTH28; FtTH4 and FtTH27*) located on the tartary buckwheat chromosomes, and all of them are located on two different chromosomes, except chromosome 4, chromosome 5 and chromosome 6, where there were no duplicated segments located on (Fig. 4).

### Evolutionary analyses and synteny analysis within *FtTH* genes and several other species

**Fig. 5:**
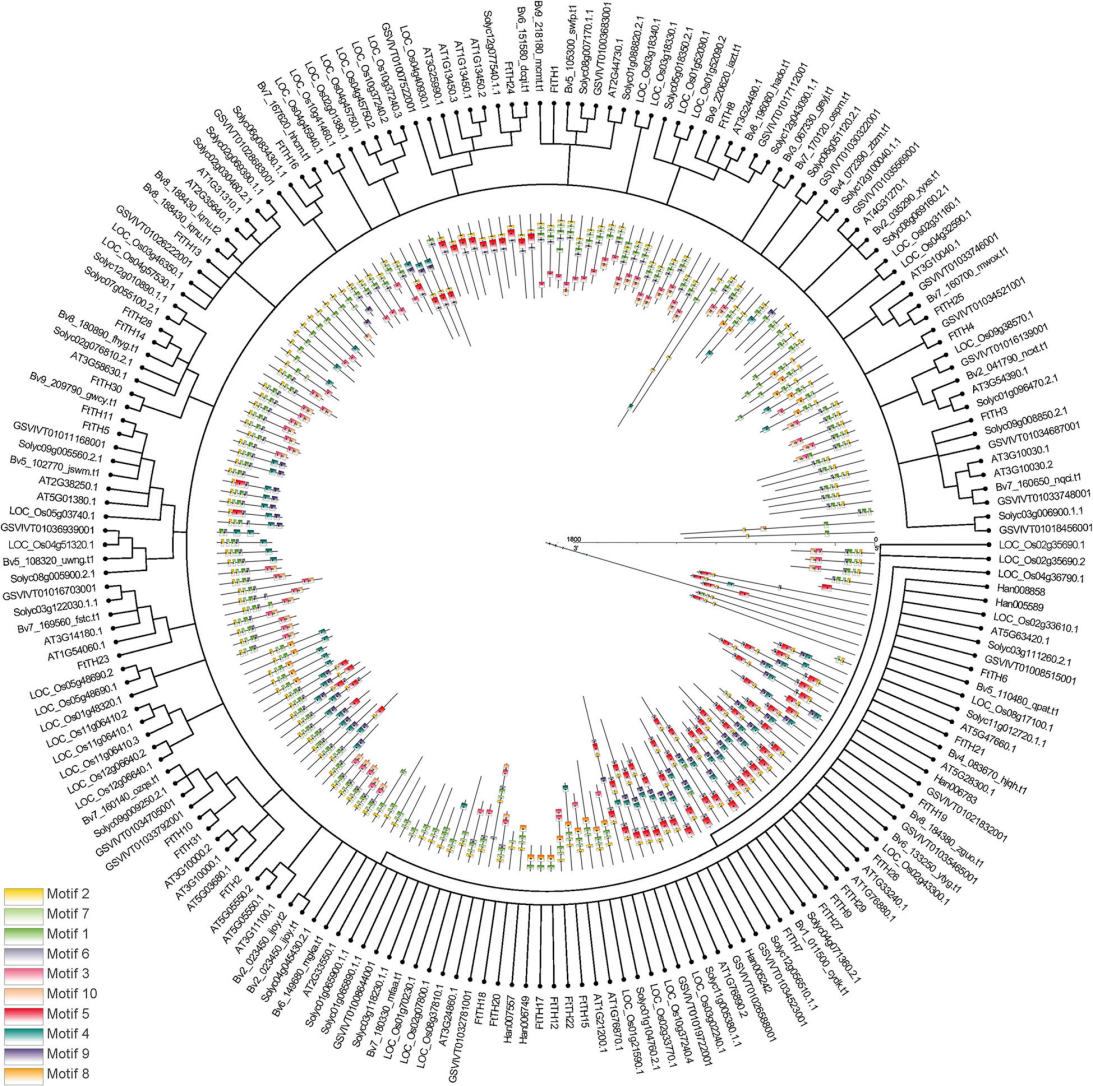
Phylogenetic relationships and motif compositions of the trihelix proteins from seven different plant species (tartary buckwheat, *Arabidopsis*, beet, tomato, grape, rice and sunflower). The motifs, numbered 1–10, are displayed in different colored boxes. The sequence information for each motif is provided in Additional file 2: Table S2

To analyze the evolution relation futures of the *trihelix* family among tartary buckwheat and six plants (*Arabidopsis*, beet, tomato, grape, rice and sunflower), an unrooted NJ tree with 10 conserved motifs was constructed using the NJ method of Geneious R11 according to the protein sequences of 31 *FtTH* genes and six other plant *trihelix* genes (Fig. 5). Detailed genetic correspondence is presented in Additional file 3: Table S3. The distribution of *FtTHs* in the phylogenetic tree is relatively dispersed. Almost all *trihelix* family members from different species shown in Fig. 5 share motif 2. Most *trihelix* family members contain motif 1 and motif 7.

**Fig. 6:**
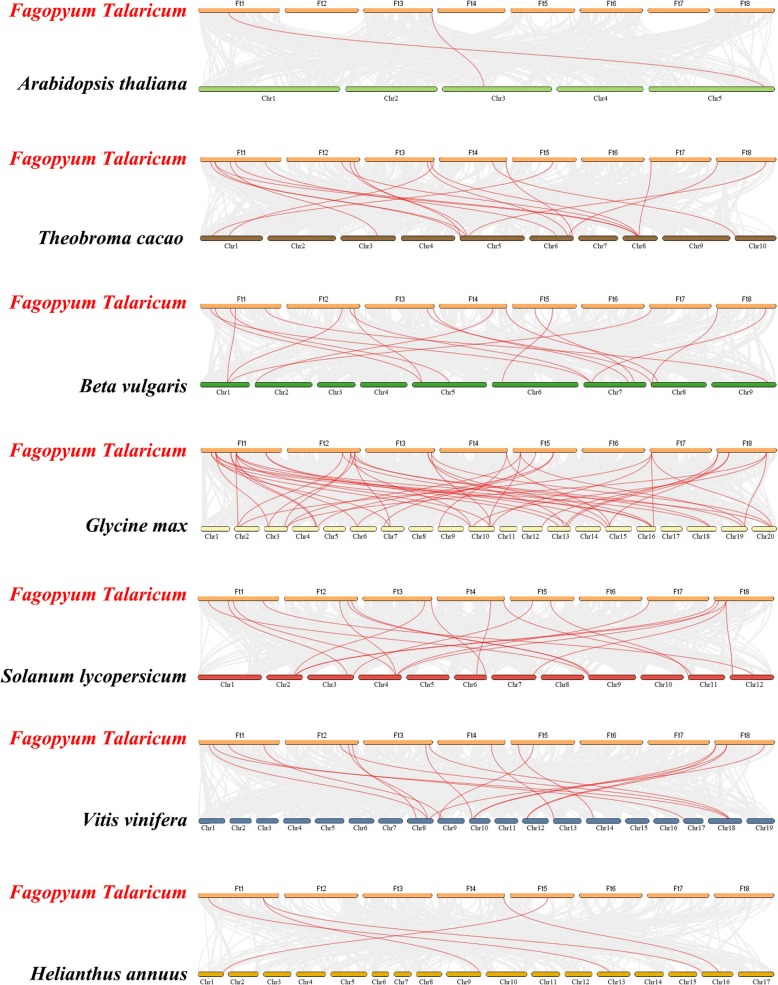
Synteny analyses between the *trihelix* genes of tartary buckwheat and seven representative plant species

To analyze the synchronization relation in the tartary buckwheat *trihelix* family, we constructed seven comparative system diagrams between tartary buckwheat and seven dicotyledonous plants (Arabidopsis, cacao, beet, soybean, tomato, grape and sunflower) as shown in Fig. 6. From the details provided in Additional file 3: Table S3, *FtTH* genes displayed syntenic relationships in different degrees with soybean (46), followed by tomato (19), cacao (18), grape (17), beet (17), sunflower (5) and *Arabidopsis thaliana* (2). Generally, tartary buckwheat was the most similar with soybean and the lowest with *Arabidopsis thaliana* through comparison in these diagrams, which might be closely associated with the phylogenetic evolutionary relationship among them.

### Expression patterns for *FtTH* genes in different tissues/organs

**Fig. 7:**
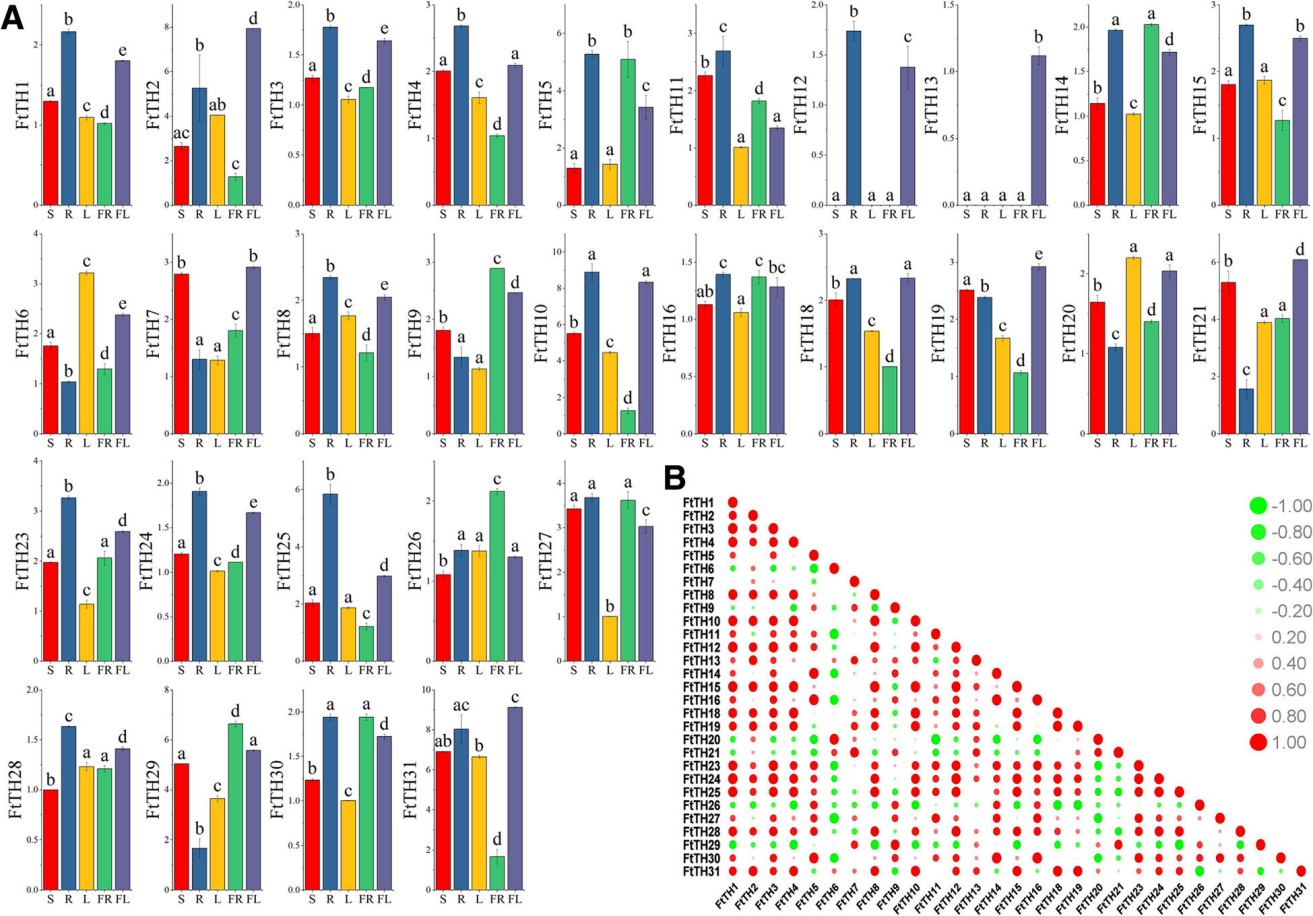
Tissue-specific gene expression of 29 tartary buckwheat *trihelix* genes and the correlation between the gene expression patterns of *FtTHs*. **a.** The expression patterns of 29 tartary buckwheat *trihelix* genes in stem (S), root (R), leaf (L), fruit (FR) and flower (FL) tissues were examined by qPCR. Error bars were obtained from three measurements. Lowercase letter(s) above the bars indicate significant differences (α = 0.05, LSD) among the treatments. **b.** The red round spot: positively correlated; the green round spot: negatively correlated. The deepest and largest red round spot indicate a significant correlation at the 0.05 level

In plants that have been studied and reported, functional studies of many genes have shown that *trihelix* genes act a key factor in crop growth and development [5]. To obtain insights into the physiological effect of the *FtTH* gene in the development of tartary buckwheat, the expression of *FtTH TF* family members in different tissues/organs was measured using qRT-PCR. In selected organs/tissues, including stem, root, leaf, fruit and flower, the expression profiles of *FtTH* genes are shown with histograms (Fig. 7a). The *FtTH* genes were expressed in all selected organs/tissue except *FtTH17 and FtTH22*, which had no expression. The result showed that the transcriptional products expressed by 29 *FtTH* genes was highly expressed in specific organs/tissues, suggesting that *FtTH* family members have diverse functions during tartary buckwheat development stages. By looking at the column height of each histogram, seven *FtTH* genes (*FtTH2, FtTH7, FtTH13, FtTH18, FtTH19, FtTH21 and FtTH31*) were expressed at high levels in tartary buckwheat flowers (Fig. 7a), and *FtTH13* was only expressed in the flower. Seventeen *FtTH* genes (*FtTH1, FtTH3, FtTH4, FtTH5, FtTH8, FtTH10, FtTH11, FtTH12, FtTH15, FtTH16, FtTH18, FtTH23, FtTH24, FtTH25, FtTH27, FtTH28 and FtTH30*) were expressed more in the root than in other organs/tissue, where interestingly, *FtTH12* was only expressed in the root and flower. *FtTH6* and *FtTH20* were highly expressed in tartary buckwheat leaves. Five *FtTH* genes (*FtTH9, FtTH14, FtTH26, FtTH29 and FtTH30)* were expressed at relatively high levels in fruit. In the tartary buckwheat stem, the expression of all *FtTH* genes was less than in other organs/tissues.

At the same time, we studied the correlation between expression profiles of these 29 *FtTH* genes (Fig. 7b). A majority of the *FtTH* genes were positively related, and especially these *FtTH* genes (*FtTH1, FtTH4, FtTH8, FtTH10, FtTH12, FtTH15, FtTH18 and FtTH24*) that were significantly correlated with many the other *FtTH* genes (Fig. 7b). While some *FtTH* genes *(FtTH6, FtTH20, FtTH21, FtTH26 and FtTH29)* were negatively correlated with most of others and the pairs of *FtTH* genes *(FtTH6 and FtTH11/ FtTH27; FtTH20* and *FtTH11)* were significantly negatively correlated with each other.

### Expression profiles of *FtTH* genes at fruit development stages of tartary buckwheat

**Fig. 8:**
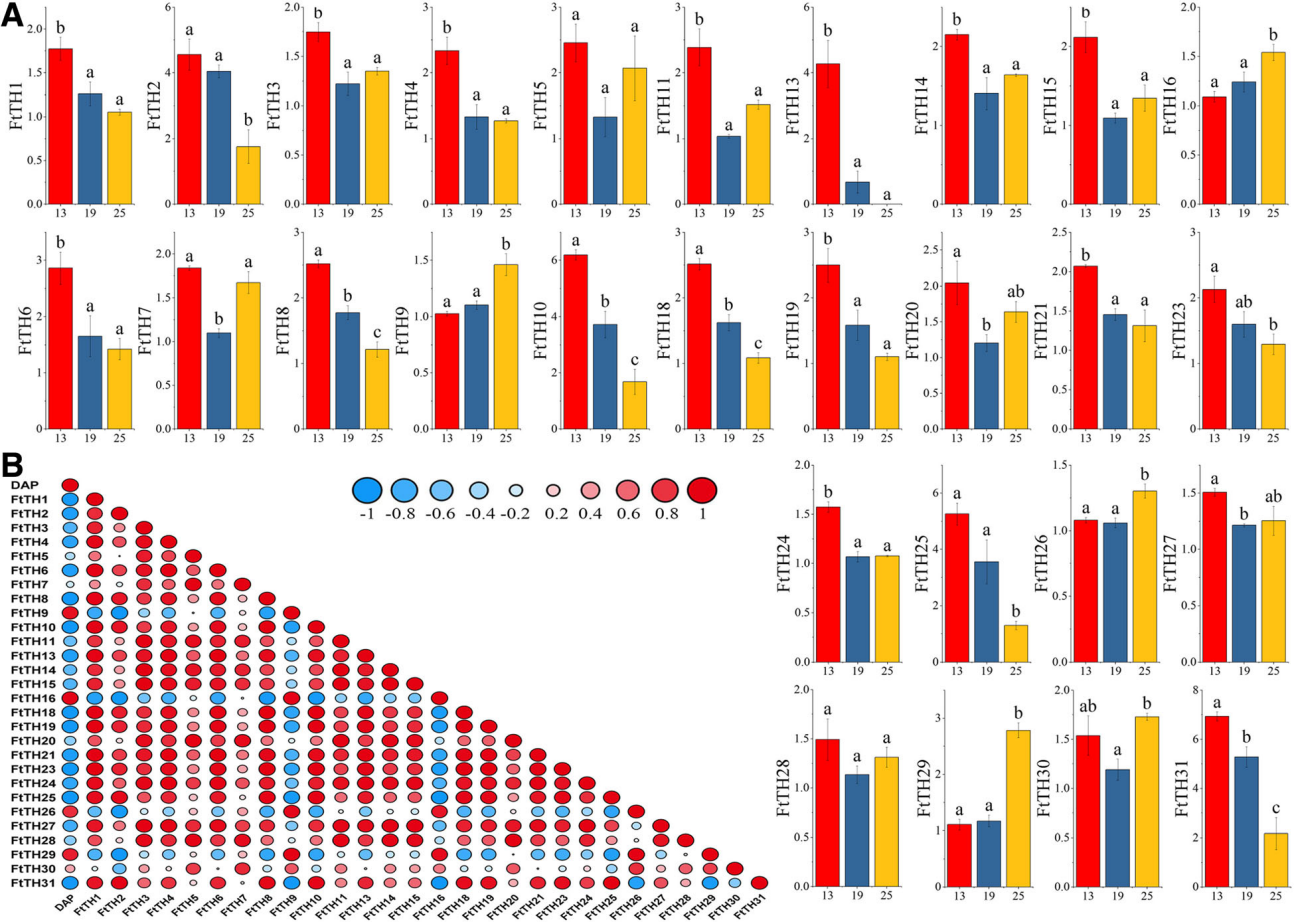
Gene expressions of 28 tartary buckwheat *trihelix* genes during fruit development and the correlation between the gene expression patterns of *FtTHs* during fruit development. **a.** The expression patterns of 28 tartary buckwheat *trihelix* genes in the fruit development stage were examined using a qRT-PCR assay. Error bars were obtained from three measurements. Lowercase letter(s) above the bars indicate significant differences (α = 0.05, LSD) among the treatments. **b.** The red round spot: positively correlated; the blue round spot: negatively correlated. The deepest and largest red round spot indicate a significant correlation at the 0.05 level

Tartary buckwheat has a high value in nutrition and medicine. The total contents of flavonoids and amino acid balance proteins are higher than those of primary food crops [38]. The fruit of tartary buckwheat is the main part of exerting its value. *Trihelix* genes regulate diverse biological processes such as embryogenesis [5]. Therefore, it is of substantial interest to study the expression of *FtTH* genes during fruit development. By analyzing the characteristics of *FtTH* gene expression, the *FtTH* genes that were closely correlated with the development of tartary buckwheat fruit were screened out. The time difference of the expression of 28 *FtTH* genes in the growth and development of tartary buckwheat fruit is presented in the form of histograms, except for *FtTH12, FtTH17 and FtTH22* (Fig. 8a). The experimental results showed that the expressed product abundance of 28 *FtTH* genes obviously varied at 13 DPA (green fruit stage), 19 DPA (discoloration stage), and 25 DPA (initial maturity stage) [61], indicating that some *FtTH* genes have substantial functions at the development stages of tartary buckwheat fruit. The level of expression of three *FtTH* genes (*FtTH9, FtTH16 and FtTH26*) increased progressively during tartary buckwheat fruit development as shown in Fig. 8a. The expression of fourteen *FtTH* genes (*FtTH1, FtTH2, FtTH4, FtTH6, FtTH8, FtTH10, FtTH13, FtTH18, FtTH19, FtTH21, FtTH23, FtTH24, FtTH25 and FtTH31)* decreased by degrees. The expression patterns of eleven others fluctuated.

In addition, most *FtTH* genes were negative related to fruit development, and *FtTH10* had a significantly negative correlation. This was consistent with the gene expression pattern during development described above. The correlation of these 28 *FtTH* gene expression patterns was analyzed in tartary buckwheat fruit. Most of the *FtTH* genes had positive correlation, and many *FtTH* genes (*FtTH3 and FtTH14/FtTH15; FtTH4 and FtTH24; FtTH6 and FtTH13/FtTH21; FtTH8 and FtTH10/FtTH18; FtTH13 and FtTH21; FtTH14 and FtTH11/FtTH15; FtTH19 and FtTH1/FtTH18; FtTH20 and FtTH28; FtTH23 and FtTH8/FtTH18/FtTH19)* were significantly positively correlated (Fig. 8b). Four *FtTH* genes (*FtTH2* and *FtTH9; FtTH16* and *FtTH31*) were significantly negatively correlated.

## Discussion

Tartary buckwheat is an important cash crop [64]. Research on the whole genome of the *trihelix* gene family in tartary buckwheat has not been reported. Zhang et al. (2017) reported the reference genome of tartary buckwheat, and Liu et al. (2018–2019) studied tartary buckwheat [52–56, 61, 65], both of which provide an abundant theoretical basis for us to systematically analyze the characteristics and functions of tartary buckwheat *trihelix*. We identified 31 *FtTH* genes (Additional file 1: Table S1), and the number of *FtTH* genes is close to that in *Arabidopsis thaliana* and rice [5]. Phylogenetic analysis showed that the tartary buckwheat *trihelix* genes were formed into 5 clades by constructing an unrooted NJ tree to analyze and compare with *trihelix* family members in *Arabidopsis thaliana* (Fig. 1). Based on the phylogenetic tree, analyzing the function of known genes is helpful to infer the function of *FtTH* family members, which has certain theoretical support for the functional exploration of candidate genes in the later stage.

We analyzed the conserved motifs and structure of the members of the *trihelix* family of tartary buckwheat (Fig. 2). The result of motifs and structure is consistent with our classification results. The similarity of most members in the same clade indicates that the conserved motifs may play a key role in the function of a particular population. The composition of the motifs of the different branches is large; therefore, the function of the tartary buckwheat trihelix protein is complex. The sequence distribution indicates that the genes containing the same motif may be produced by the amplification of genes in the same population. This is similar to the report in chrysanthemums [66]. Among five clades, GT-1 and GT-2 were studied relatively early, and the homology between them was much higher than that of other subfamilies [5]. The high similarity of the conserved motifs composition in GT-1 and GT-2 (most members of both clades share motif 1, 2, 3, 6, 7 and 8) coincides with this statement.

Gene replication plays an important role in gene functional diversity and gene amplification, and is one of the most important factors of biological evolution, including tandem replication, segmental replication, and genomic replication [67]. Compared with soybean (67), *Populus trichocarpa* (56) and *Brassica Rapa* (52), the number of *FtTH* genes from tartary buckwheat is less [16, 18, 19]. The difference may be due to the σ genome duplication (WGD) event happening in other species after their earliest ancestors differentiated, but not in tartary buckwheat [63]. In addition, gene replication can amplify the number of genes. One of the main reasons for the amplification of many gene families is the segmental replication [63]. According to the results, only segmental duplication events were discovered without tandem duplication events (Figs. 3 and 4). There were 8 pairs of segmental duplication on 5 tartary buckwheat chromosomes. We hypothesize that the emergence of some *FtTH* genes and the evolution of *FtTH* genes are probably driven by these segmental duplication events. This is similar to research in *Populus trichocarpa* [19].

According to the result of chromosomal distribution, chromosome 6 harbors no *FtTH* gene, indicating that *FtTH* gene family may have suffered gene loss during a long evolutionary process [68]. In addition, there were segmental replication events but no tandem replication events in *FtTH* gene family of tartary buckwheat. Gene replication plays an important role in genome amplification and recombination [69]. High segmental replication rate is beneficial to gene evolution. The loss of some *FtTH* genes may be due to the dynamic changes after segmental replication, which is consistent with the results in the *Populus trichocarpa* [19].

The gene expression pattern is an important factor in judging the function and characteristics of the *trihelix TF* family, so we determined the expression of genes in tartary buckwheat stem, root, leaf, fruit and flower by qRT-PCR. As shown in the histograms in Fig. 7a, the expression characteristics of *FtTH* genes showed the spatial variation of expression. The *trihelix* gene family plays a role in plant growth and development processes [5]. By analyzing the expression characteristics of some *FtTH* family members in specific plant tissues/organs, we can hypothesize that *FtTH TF* plays a specific and significant role in tartary buckwheat growth and development. Most of the *FtTH* family members have wide expression in the tartary buckwheat tissues/organs and two *FtTH* genes (*FtTH12* and *FtTH13*) present a tissue-specific expression pattern. Tissue-specific genes may play a role in the growth and differentiation of the corresponding organs or tissues, but more experiments are needed to verify the function of these *FtTH* genes [66]. Under drought conditions, the root system can perceive the change of soil and transmit a series of signals to the leaves of the plant, thus triggering the physiological and biochemical reaction of the plant to reduce the damage to the root system [70]. Four *FtTH* genes (*FtTH4, FtTH12, FtTH23 and FtTH25*) that are expressed significantly highly in roots, may contribute to the adaptation of tartary buckwheat to arid conditions. This is consistent with the nature of tartary buckwheat as a drought-tolerant crop. Similar conclusions have been drawn in poplars [19]. In the context of abiotic stress, some studies have shown that *AtGT2L (At5g28300),* a member of the Arabidopsis GT-2 subfamily that is highly expressed in petals and leaves of *Arabidopsis thaliana*, is responsive to cold stress and drought stress, and its expression increased [31]. *FtTH21* of GT-2 homologous to *AtGT2L* has high expression in tartary buckwheat flowers. *FtTH21* can be used as a candidate gene, and future experiments can be conducted to verify whether it can adapt to drought and cold by changing expression levels. In addition, *GTL1 (At1g33240)* belongs to the GT-2 subfamily and is related to the stomatal number of leaves. *GTL1* can directly inhibit the expression of *STOMATAL DENSITY AND DISTRIBUTION1 (SDDI)* gene. A serine protease encoded by *SDDI* plays a negative role in stomatal regeneration. Thus, the *gtl1* mutants showed a decrease in the number of stomata in leaves. Under drought conditions, the expression of *GTL1* decreases, which contributes to water use because plants with fewer stomata can reduce water loss [28, 32]. *FtTH6* grouped in tartary buckwheat GT-2 with *GTL,* has a relatively high expression in the leaf, and most likely regulates water use by changing the stomatal density. Tartary buckwheat is a drought-tolerant species. This function coincides with the physiological characteristics of tartary buckwheat. In the SIP1 subfamily, *At3g10030* is related to leaf development; the corresponding mutant of this gene is short and leaves are deformed and discolored [71]. *FtTH3* homologous to *At3g10030* exhibited the lowest levels in tartary buckwheat leaves and may be related to the normal development of leaves. *PETAL LOSS (PTL),* also designated *At5g03680*, is a *trihelix* family member from GT-2 related to the development of petals and sepals [25–27]. PTL protein can inhibit the growth of sepals so that the sepals do not fuse together. *Ptl* mutants show petal reduction and sepal fusion [27]. In addition, PTL activates RABBIT EARS (RBE) and promotes the growth of petals [72]. Therefore, in flowering plants, *PTL* plays a significant role in the normal formation and growth of flowers. *FtTH2 and FtTH31* from GT-2, which exhibited relatively high expression in tartary buckwheat flowers, are homologous to *PTL.* These two genes may function in controlling the initiation and development of the petals of tartary buckwheat flowers, which can be further verified by later experiments.

To further explore the function of *FtTH* genes in the fruit development (13, 19, 25 DPA) of tartary buckwheat, we determined the expression of *FtTH* genes during fruit development by qRT-PCR. Their expression profiles showed time differences. In the GT-1 subfamily, *EMB2746 (At5g63420),* which exists in *Arabidopsis thaliana*, is widely expressed in the vegetative part of *Arabidopsis thaliana*, especially in seeds. The seeds of the *emb2746* mutant only develop to the globular stage; therefore, *EMB2746* is necessary for early embryogenesis. *LOC_Os02g33610* in rice is also similar to *At5g63420* [73]. *FtTH6,* which is homologous to *At5g63420* (Fig. 1) and *LOC_Os02g33610* (Fig. 5), is expressed in all selected tissues/organs (Fig. 7a) and has a relatively higher expression at 13 DPA (green fruit stage) in fruit, as shown in Fig. 8a. *FtTH6* may also play an equally important role in the early development and normal development of tartary buckwheat fruits. In addition, *ASIL1 (At1g54060)* from the SIP1 subfamily plays a significant role during the transition from vegetative growth to reproductive growth. In seedlings, the *ASIL1* transcription product combines with the inhibitory element (GTGATT) in the *2S3* promoter of the seed storage protein gene to avoid the synthesis and accumulation of seed similar storage substances, such as seed specific storage protein 2S3, in the seedling. *ASIL1* helps to control seed maturation at the appropriate stage of development [74–76]. *FtTH18* and *FtTH23* of SIP1 were homologous to these genes and expressed at relatively low levels in tartary buckwheat fruit (Fig. 7a). In particular, the expression of *FtTH18* was the lowest in fruit compared with other tissues. The expression patterns of these two *FtTH* genes decreased in fruit development stages (Fig. 8a). *FtTH18* and *FtTH23* have similar functions. The decreased expression of the two *FtTH* genes indicates that with the decrease of the expression of *FtTH18* and *FtTH23*, the inhibitory effect of these two genes on the mature genes of seeds was weakened, and the fruit of tartary buckwheat accumulated protein and other storage materials normally with the embryogenesis stage, which made the fruit develop and mature normally. The timing accuracy of fruit maturation is regulated. The discussion provides a basis for the later experiment to verify the similarity of functional characteristics of the *FtTH* gene and *Arabidopsis thaliana trihelix* in the fruit growth and development of tartary buckwheat in the future.

## Conclusions

Our first step was to conduct a genome-wide analysis to identify and analyze the *FtTH TF* family in tartary buckwheat. We identified 31 *FtTH* genes and analyzed their physical properties, evolutionary relationships, gene structures and replication. The expression patterns of *FtTH TFs* in normal growth conditions were analyzed by qRT-PCR. Based on the above discussion and hypotheses of the functional characteristics of the *FtTH* family, *FtTH* genes play significant roles during tartary buckwheat development. We initially screened out some of the key candidate genes, which provides a theoretical basis for us to further explore the functional characteristics in tartary buckwheat through experiments and improve crop yield of tartary buckwheat.

## Additional files

Additional file 1: **Table S1.** List of the 31 *FtTH* genes identified in this study. (XLS 120 kb)

Additional file 2: **Table S2.** Analysis and distribution of the conserved motifs in tartary buckwheat trihelix proteins. (XLS 33 kb)

Additional file 3: **Table S3.** One-to-one orthologous relationships between tartary buckwheat and other plants. (XLS 99 kb)

Additional file 4: **Table S4.** The primer sequences for qRT-PCR. (XLS 36 kb)

## Data Availability

The genome sequences of tartary buckwheat used for identifying the *trihelix* genes in this study were located in the Tartary Buckwheat Genome Project (TBGP; http://www.mbkbase.org/Pinku1/). The tartary buckwheat accession (XIQIAO) materials used in the experiment were supplied by Professor Wang Anhu of Xichang University. The datasets supporting the conclusions of this article are included in the article and its Additional files.

## Abbreviations

Aa: Amino acid
CDS: Coding sequence length
DPA: Days postanthesis
FtTH: Fagopyum talaricum trihelix
GSDS: Gene Structure Display Server
HMM: Hidden Markov Model
LSD: Least significant difference
MCScan: Multiple collinear scanning
MW: Molecular weight
NJ: Neighbor-Joining
Pfam: Protein family
PI: Isoelectric point
PTL: PETAL LOSS
qRT-PCR: Real-time quantitative PCR
RBE: RABBIT EARS
SDDI: STOMATAL DENSITY AND DISTRIBUTION1
TBGP: Tartary Buckwheat Genome Project
TF: Transcription factor
UV-b: Ultraviolet-b
